# Neuroprotective and Antioxidant Properties of CholesteroNitrone ChN2 and QuinolylNitrone QN23 in an Experimental Model of Cerebral Ischemia: Involvement of Necrotic and Apoptotic Cell Death

**DOI:** 10.3390/antiox12071364

**Published:** 2023-06-29

**Authors:** Beatriz Chamorro, Sara Izquierdo-Bermejo, María Dolores Martín-de-Saavedra, Francisco López-Muñoz, Mourad Chioua, José Marco-Contelles, María Jesús Oset-Gasque

**Affiliations:** 1 Department of Biochemistry and Molecular Biology, Faculty of Pharmacy, Complutense University of Madrid, Plaza Ramón y Cajal s/n, Ciudad Universitaria, 28040 Madrid, Spain; beatrcha@ucm.es (B.C.); sizqui03@ucm.es (S.I.-B.); marmar68@ucm.es (M.D.M.-d.-S.); 2 Faculty of Health, Camilo José Cela University, Villanueva de la Cañada, 28692 Madrid, Spain; flopez@ucjc.edu; 3 Instituto Universitario de Investigación en Neuroquímica, Complutense University of Madrid, Ciudad Universitaria, 28040 Madrid, Spain; 4 Neuropsychopharmacology Unit, “Hospital 12 de Octubre” Research Institute, 28041 Madrid, Spain; 5 Laboratory of Medicinal Chemistry, Institute of Organic Chemistry (CSIC), C/Juan de la Cierva 3, 28006 Madrid, Spain; mchioua@gmail.com (M.C.); jlmarco@iqog.csic.es (J.M.-C.); 6 Center for Biomedical Network Research on Rare Diseases (CIBERER), Carlos III Health Institute (ISCIII), 28029 Madrid, Spain

**Keywords:** cerebral ischemia, neuroprotection, nitrones, oxidative stress, stroke, therapeutic agents

## Abstract

Ischemic stroke is the leading cause of disability and the second leading cause of death worldwide. However, current therapeutic strategies are scarce and of limited efficacy. The abundance of information available on the molecular pathophysiology of ischemic stroke has sparked considerable interest in developing new neuroprotective agents that can target different events of the ischemic cascade and may be used in combination with existing treatments. In this regard, nitrones represent a very promising alternative due to their renowned antioxidant and anti-inflammatory effects. In this study, we aimed to further investigate the neuroprotective effects of two nitrones, cholesteronitrone 2 (**ChN2**) and quinolylnitrone 23 (**QN23**), which have previously shown great potential for the treatment of stroke. Using an experimental in vitro model of cerebral ischemia, we compared their anti-necrotic, anti-apoptotic, and antioxidant properties with those of three reference compounds. Both **ChN2** and **QN23** demonstrated significant neuroprotective effects (EC_50_ = 0.66 ± 0.23 μM and EC_50_ = 2.13 ± 0.47 μM, respectively) comparable to those of homo-bis-nitrone 6 (**HBN6**) and N-acetylcysteine (**NAC**) and superior to those of α-phenyl-N-tert-butylnitrone (**PBN**). While primarily derived from the nitrones’ anti-necrotic capacities, their anti-apoptotic effects at high concentrations and antioxidant powers—especially in the case of **QN23**—also contribute to their neuroprotective effects.

## 1. Introduction

Stroke is a is a medical condition caused by the disruption of blood flow to one or several brain regions [1]. It is currently the leading cause of disability and the second leading cause of death worldwide [2]. Its incidence rate continues to grow, driven by the increase in life expectancy in developed countries [3] and the accumulation of risk factors in increasingly younger populations [1,4].

Ischemic stroke involves a complex set of molecular processes at the biochemical level, known as the ischemic cascade, which results from cellular bioenergetic dysfunction. These processes include glutamate excitotoxicity events [2,5,6], oxidative stress [5,7,8], neuroinflammation [2,5], blood–brain barrier (BBB) dysfunction [2,5,9], and alterations in Ca^2+^ homeostasis [5,6]. All these neuropathological events trigger a series of signaling cascades that induce programmed cell death via apoptosis and autophagy, mainly in the penumbra, and unprogrammed cell death via necrosis, primarily at the core [5,10,11,12].

Despite its high social and economic costs, therapeutic strategies aimed at treating ischemic stroke are limited in both their availability and effectiveness. Current treatment primarily relies on the restoration of cerebral perfusion using the recombinant tissue plasminogen activator (rtPA), a thrombolytic drug that has many limitations, including a very narrow therapeutic window and many exclusion criteria that result in only 2–5% of patients receiving it [10,13,14]. However, with the abundance of information currently available on the molecular pathophysiology of this disease, there is great interest in developing new neuroprotective agents that target various events of the ischemic cascade [15,16]. These agents aim to reduce brain damage during ischemia and reperfusion, act as adjuvants and extend the therapeutic window of thrombolytic therapy, and achieve functional improvements that reduce the severity of sequelae [1,15,16].

Nitrones are among these neuroprotective agents. They are organic molecules with antioxidant [4,10] and anti-inflammatory [17] properties that have also been used as starting molecules for the synthesis of hybrid compounds with synergetic effects, such as cholesteronitrones [4,18]. Clinical trials for some nitrone compounds, such as **NXY-059**, have been conducted, but their neuroprotective potential in laboratory experiments has not yet been translated to the clinic [1,19]. One possible reason for this could be the limitations of the mouse models for ischemic stroke and the evaluation methods [1,6,14], as well as the many complications involved in designing clinical trials for treating stroke [6,13,16].

Our research group has been actively working on the synthesis and pharmacological characterization of new nitrones for the treatment of ischemic stroke [4,10,18,19,20,21,22,23]. To date, we have conducted research on the neuroprotective effects of cholesteronitrones [4,18], quinolylnitrones [20,21,22] and, more recently, α-phenyl-N-tert-butylnitrone (**PBN**)-derived bis-nitrones [10] and tris-nitrones [23]. Among the cholesteronitrones and quinolylnitrones studied, cholesteronitrone 2 (**ChN2**) and quinolylnitrone 23 (**QN23**) have shown outstanding potential due to their ability to decrease brain lesion size. **ChN2** reduces the infarct size by 21%, while **QN23** lowers it to 44% when administered in mice at certain doses (0.05–1 mg/kg of **ChN2** and 1.5–2 mg/kg of **QN23**) after undergoing transient middle cerebral artery occlusion (tMCAO) [4,20]. In addition, these compounds have been shown to improve neurological function and promote motor capacity recovery in animal models of ischemic stroke [4,20,22]. Another promising nitrone is homo-bis-nitrone 6 (**HBN6**), which reduced infarct size by up to 90% when administered to mice that underwent permanent middle cerebral artery occlusion (pMCAO) [10]. In addition to their in vivo efficacy, these three nitrones exhibit great neuroprotective and antioxidant potential in in silico and in vitro models. **ChN2** and **QN23** act as free radical scavengers [18,20] and increase cell viability, reducing apoptosis in neuronal primary cultures [18] and in mouse brain slices [4]. In particular, **HBN6** is capable of reversing the decrease in neuronal metabolic activity caused by ischemia (EC_50_ = 1.24 ± 0.39 μM), as well as acting as a potent hydroxyl radical scavenger in silico [10]. In addition, **HBN6** reduces superoxide anion production in cultures of human neuroblastoma cells (EC_50_ = 5.91 ± 1.09 μM and maximal activity = 95.8 ± 3.6%) [10].

**Figure 1:**
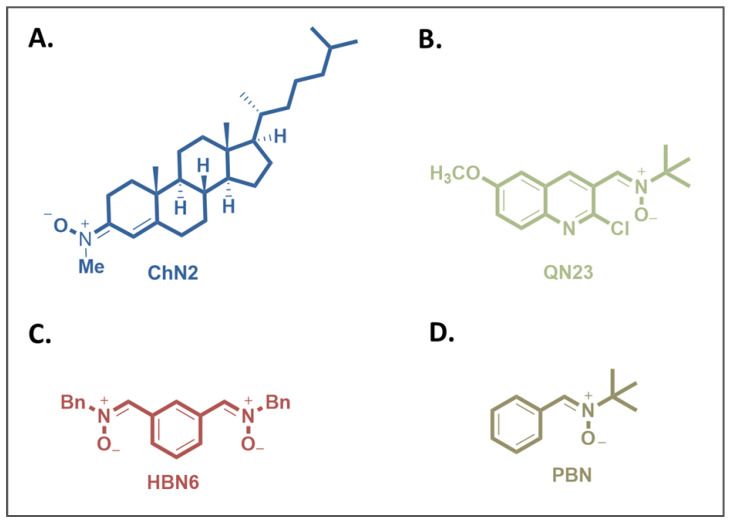
Structures of (**A**) cholesteronitrone **ChN2**, (**B**) quinolylnitrone **QN23**, (**C**) homo-bis-nitrone 6 (**HBN6**), and (**D**) α-phenyl-N-tert-butylnitrone (**PBN**).

Given the promising protective properties of these nitrones in cerebral ischemia models, we aimed to conduct a more comprehensive investigation of their neuroprotective properties. We selected **ChN2** (Figure 1A) and **QN23** (Figure 1B) as they have shown the greatest therapeutic potential in both in vitro and in vivo studies. Our goal was to comparatively analyze the contributions of the inhibition of necrosis and apoptosis and of the production of reactive oxygen species (ROS) to the neuroprotective effects of these nitrones against cerebral ischemia. We also juxtaposed their effectiveness against that of **HBN6** (Figure 1C) (a nitrone developed by our research group and used as an internal reference) and two standards with proven efficacy, namely, **PBN** (Figure 1D) (the parent nitrone that provides their structural base) and *N*-acetylcysteine (**NAC**), a well known antioxidant [3].

## 2. Materials and Methods

### 2.1. Neuroblastoma Cell Cultures

SH-SY5Y human neuroblastoma cells (ATCC CRL-2266) were cultured in flasks containing Dulbecco’s/Ham’s F12 (Gibco, Life Technologies, Madrid, Spain) 1:1 (*v*/*v*) with 2.5 mM of GlutaMAX^TM^, (Gibco, Life Technologies, Madrid, Spain), 1% antibiotic–antimycotic (100 mg/mL of streptomycin, 100 µL/mL of penicillin, and 0.25 mg of amphotericin B) (Gibco, Life Technologies, Madrid, Spain), 1% gentamicin, 15 mg/mL (Sigma-Aldrich, Madrid, Spain), and 10% fetal bovine serum (FBS) (Gibco, Life Technologies, Madrid, Spain) as previously described [10,21,23]. The cultures were maintained at 37 °C in a humidified atmosphere of 5% CO_2_ and 95% air. The culture medium was changed every 2 days, and upon reaching confluence, the cells were subcultured into new flasks after undergoing partial digestion with 0.25% trypsin-EDTA (Gibco, Life Technologies, Madrid, Spain). For each experiment, the SH-SY5Y cells were seeded in 48- or 96-well plates at densities of 1–1.5 × 10^5^ cells/well or 0.5 × 10^5^ cells/well, respectively.

#### 2.1.1. Treatment of Neuroblastoma Cell Cultures with Oligomycin–Rotenone (O-R)

To induce oxidative stress, SH-SY5Y cells were treated with oligomycin and rotenone, inhibitors of complexes I and V of the respiratory chain, respectively. A mixture of 10 μM of oligomycin and 30 μM of rotenone was added to the wells, followed by the addition of various concentrations of the tested compounds (0.001–1000 μM). The plates were then kept for 24 h in an aerobic culture chamber (95% air and 5% CO_2_) before the cell viability assays were performed. A control condition was included, maintaining cells in standard Dulbecco’s medium without adding O-R or any neuroprotective compound. Additionally, vehicle-only control conditions were included, employing ethanol (the solvent used for **ChN2**) and dimethyl sulfoxide (the solvent used for the rest of the molecules). 

#### 2.1.2. Neuroblastoma Cell Cultures’ Exposure to Oxygen–Glucose Deprivation (OGD) 

To induce experimental ischemia (I), SH-SY5Y neuroblastoma cells were subjected to OGD for 3 h. This was achieved by adding glucose-free Dulbecco’s medium (Gibco, Life Technologies, Madrid, Spain) to the wells and placing the plates inside an anaerobic chamber in the presence of a gas mixture of 95% N_2_/5% CO_2_ which was humidified at 37 °C and at a constant pressure of 0.15 bar. After the OGD period, the glucose-free medium was replaced by the standard oxygenated medium oxygen, the compounds were added to the wells at concentrations ranging from 0.001 to 1000 μM, and the cells were maintained in normoxic conditions for 24 h to simulate reperfusion (IR). It is important to emphasize that although the term “ischemia–reperfusion” (IR) is used in this paper as an abbreviation, a more accurate description when using a cellular model would be “oxygen and glucose resupply” (OGD-R). Controls were performed by preserving SH-SY5Y cells in glucose-containing Dulbecco’s medium in an incubator in normoxic conditions for 3 h, after which the medium was changed, and the cells were kept in the same conditions for an additional 24 h. Sodium nitroprusside (SNP) was also included at concentrations of 2 mM and 5 mM as a positive control for necrotic and apoptotic cell death. Moreover, we included the same concentrations of the vehicle used to dissolve the tested compounds (final concentration < 1%) in all experiments.

### 2.2. Evaluation of Cell Viability

To assess the neurotoxicity and neuroprotective effects of the compounds, cell viability was measured in SH-SY5Y human neuroblastoma cells seeded in 96-well culture plates at a density of 0.5 × 10^5^ cells/well. After subjecting the cells to O-R, OGD-R, or a control treatment, **ChN2**, **QN23**, **HBN6**, **PBN**, and **NAC** were added at concentrations ranging from 0.001 to 1000 µM. Then, cell viability was evaluated using the Cell Proliferation Kit II (XTT) (Sigma Aldrich, Madrid, Spain), which quantifies cellular respiration based on the ability of metabolically active cells to reduce a yellow tetrazolium salt to an orange formazan dye [23]. After incubating the cells with an XTT solution (0.3 mg/mL) for 2 h at 37 °C with 5% CO_2_ and 95% air (*v*/*v*), the absorbances at 450 nm and 650 nm used as references were measured with a spectrophotometer (Power-Wave XS microplate-reader, BioTek Instruments, Madrid, Spain) [21,23]. A viability of 100% was set by the control normoxic cells treated only with standard Dulbecco’s medium. The effects of **ChN2**, **QN23**, **HBN6**, **PBN** and **NAC** were analyzed in four independent experiments, each carried out in triplicate.

### 2.3. Assessment of LDH Activity

To determine the neuroprotective effects of the compounds against necrotic cell death, SH-SY5Y cells were cultured in 48-well plates at a density of 1–1.5 × 10^5^ cells/well and exposed to OGD-R conditions (including ischemia and reperfusion controls), adding the tested compounds at 0.001–1000 µM. To assess extracellular lactate dehydrogenase (LDH) levels, media from every well were collected and stored at −20 °C until measurement. Moreover, a lysis buffer containing 0.5% Triton X-100 in 0.1 M of phosphate buffer at a pH of 7.5 was added to each well, and the cells were scratched from the bottom. These samples were centrifuged at 13,000 rpm, and intracellular LDH levels were measured in the supernatants. All measurements were obtained using a spectrophotometer reader (Power-Wave XS microplate-reader, BioTek Instruments, Madrid, Spain), and the LDH activity was calculated as the degree of absorbance decline at 340 nm, indicating the oxidation of NADH to NAD^+^, as described elsewhere [10,21]. The results were expressed as LDH activity in the extracellular medium normalized to the intracellular LDH activity obtained after cell lysis. The anti-necrotic effects of **ChN2**, **QN23**, **HBN6**, **PBN**, and **NAC** were analyzed in four independent experiments, and each experiment was carried out in triplicate.

### 2.4. Measurement of Caspase-3 Activity

To evaluate the anti-apoptotic effects of the drugs, human neuroblastoma cells were seeded in 48-well plates at a density of 1–1.5 × 10^5^ cells/well and subjected to OGD-R or standard glucose and oxygen levels as previously described. After a 24 h recovery period in normoxic conditions, the compounds were added at concentrations ranging from 0.001 to 1000 µM. The cells were then lysed in a buffer containing 5 mM of Tris/HCl (pH 8.0), 20 mM of ethylenediaminetetraacetic acid, and 0.5% Triton X-100, and the lysates were centrifuged at 13,000 rpm for 10 min. The caspase-3 activity was assessed by using the caspase substrate *N*-Acetyl-Asp–Glu–Val–Asp-amido-4-methylcoumarin (Ac-DEVD-AMC) (Merck, Madrid, Spain), which generates a fluorogenic compound after it is metabolized. Fluorescence was measured using a spectrofluorometer (Bio-Tek FL 600, BioTek Instruments, Madrid, Spain), using excitation at 360 nm and emission at 480 nm as previously described [10,21,23]. The Bradford assay was used to determine protein levels prior to the full assessment of caspase-3 activity. The anti-apoptotic effects of **ChN2**, **QN23**, **HBN6**, **PBN**, and **NAC** were evaluated in five independent experiments carried out in triplicate.

### 2.5. Determination of Reactive Oxygen Species Formation

To evaluate the antioxidant effects of the compounds, SH-SY5Y cells were seeded in 48-well plates at a density of 1–1.5 × 10^5^ cells/well. The cultured cells were subjected to OGD for 3 h, after which the glucose-free medium was replaced with oxygenated Dulbecco’s medium. Then, the compounds were added at concentrations ranging from 0.001 to 1000 µM, and the plates were maintained in normoxic conditions at 37 °C for 2.5 h. Dihydroethidium (Hydroethidine) (DHE (HEt; Molecular Probes, ThermoFisher Scientific, Madrid, Spain)), a fluorogenic molecule that reacts with the superoxide radical anion, was added to the medium, and fluorescence was recorded every 15–30 s over a 20 min period via a spectrofluorometer (Bio-Tek FL 600, BioTek Instruments, Madrid, Spain). the wavelengths of excitation and emission were 535 and 635 nm, respectively [21]. The fluorescence data were fitted using linear regression (expressed in arbitrary fluorescence units, AFUs), and the slopes were deemed indicators of mitochondrial superoxide production, as described previously [10,23,24]. The antioxidant effects of the tested compounds were analyzed in four independent experiments, and each experimental condition was tested in triplicate. 

### 2.6. Statistical Analyses

The data obtained from cell cultures were expressed as the means ± SEMs of the results from at least three independent experiments, each performed in triplicate. Statistical comparisons among the different experimental groups were performed using a one-way analysis of variance (ANOVA), followed by a Holm–Sidak post hoc test. A *p*-value < 0.05 was considered statistically significant. The fitting curves for the effective dose 50 (EC_50_) and maximal activity determinations were generated using SigmaPlot v.11 (Systat Software INC., Palo Alto, CA, USA, 2012). Curve adjustments to estimate the EC_50_ values were carried out via a non-linear regression analysis of minimal squared using logistic curves f1 = min + (max − min)/(1 + (x/EC50)^(−Hillslope)). Regression analyses and statistics were carried out via the Pearson correlation test, also using SigmaPlot v.11 (Systat Sofware INC., Palo Alto, CA, USA, 2012).

## 3. Results

This study aimed to increase our understanding of the neuroprotective and antioxidant effects of cholesteronitrone 2 (**ChN2**) and quinolylnitrone 23 (**QN23**) in SH-SY5Y human neuroblastoma cell cultures. This cell line is widely used as an in vitro model to investigate neuronal damage in ischemic stroke [25,26] and neurodegenerative diseases [27,28]. 

### 3.1. Basal Neurotoxicities of **ChN2**, **QN23**, **HBN6**, **PBN**, and **NAC**

**Figure 2:**
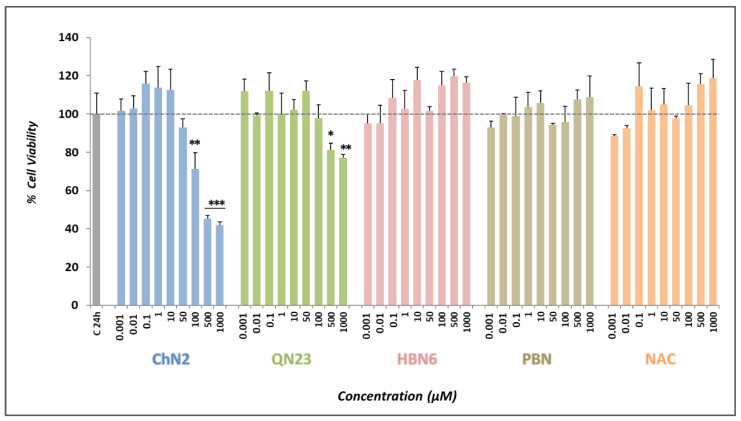
**Effect of ChN2, QN23, HBN6, PBN**, **and NAC on SH-SY5Y human neuroblastoma cell viability under basal conditions.** The bars represent the % of cell viability at the indicated concentrations of the compounds. The viability of untreated cells (C24h) was set at 100% (100 ± 10.8%; mean ± SEM). Data are shown as means ± SEMs of four experiments performed in triplicate. The statistical analysis (one-way ANOVA) reveals significant differences in viability compared to C24h (* *p* < 0.05; ** *p* < 0.01; *** *p* < 0.001). Statistical analysis of results exceeding 100% is not displayed.

As a prior step to correctly determining the neuroprotective effects of the tested compounds in experimental models of ischemia and oxidative stress, we set out to determine their basal neurotoxicities in the absence of any toxic insult via the XTT assay. While **ChN2** showed an increase in cell death at a starting concentration of 100 μM, **QN23** displayed toxic effects at concentrations of 500 μM and higher (Figure 2). At 1 mM, **ChN2** and **QN23** showed reductions in cell viability of approximately 50% and 20%, respectively. Consequently, the basal neurotoxicities of these two nitrones were considered when evaluating the results of the neuroprotection assays. On the other hand, no substantial neurotoxic effects were observed for the three other compounds used as standards (**HBN6**, **PBN**, and **NAC**) at any of the tested concentrations. These data indicate the compounds’ lack of neurotoxic effects at a wide range of concentrations, making them suitable for subsequent neuroprotective assays. Their potential in neuroprotective studies is further supported by their compatibility with the typical concentration range used in compound screening assays for hit discovery (1–10 µM).

### 3.2. Neuroprotective Profiles of **ChN2**, **QN23**, **HBN6**, **PBN**, and **NAC**

#### 3.2.1. Effect on Cell Viability

Upon confirming the lack of toxic effects of **ChN2** and **QN23** within drug discovery concentration ranges, we proceeded to investigate the neuroprotective properties of the tested compounds. We employed two in vitro models: an oxidative stress model (O-R) involving the treatment of cells with oligomycin and rotenone, and an ischemia–reperfusion model (IR) wherein cells were subjected to OGD followed by reperfusion. Both models depict events within the recanalization process, which is particularly relevant at present as it represents the ideal timeframe for the intervention of neuroprotective agents in the treatment of ischemic stroke [15,16]. An XTT assay was used to evaluate cellular metabolic capacity after exposure to the toxic insults and the addition of the compounds under study.

**Figure 3:**
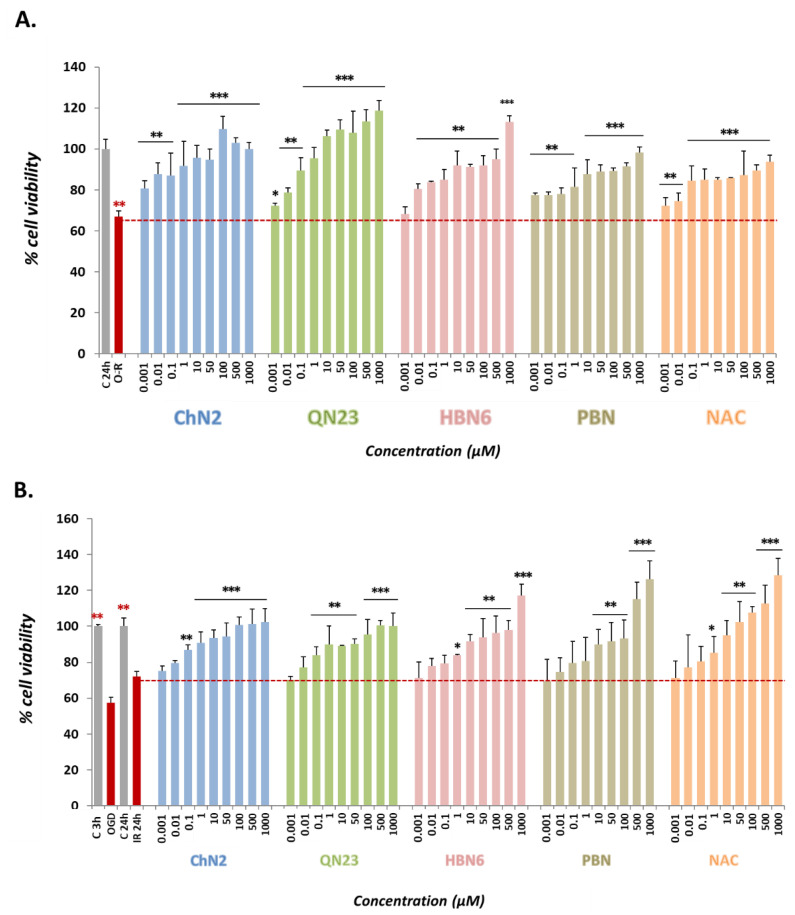
**Neuroprotective effects of ChN2, QN23, HBN6, PBN, and NAC against metabolic capacity loss induced by (A) oligomycin–rotenone (O-R) treatment and (B) oxygen–glucose deprivation (OGD) (3 h) followed by oxygen and glucose resupply (OGD-R), indicated as IR (24 h) in SH-SY5Y human neuroblastoma cells.** The bar graphs represent the % of cell viability at the in-dicated compound concentrations. Values shown are the means ± SEMs of four experiments per-formed in triplicate. Black asterisks (*): significant differences in viability between the compounds and the exposure to the O-R/ischemia–reperfusion (IR) conditions only. Red asterisks (*): significant differences in cell viability between O-R/IR models and controls (C3h and C24h) (* *p* < 0.05; ** *p* < 0.01; *** *p* < 0.001; one-way ANOVA followed by Holm–Sidak post hoc test).

After O-R treatment, a reduction in metabolic activity was observed (33.10 ± 2.90% decrease in cell viability, mean ± SEM; n = 4) (Figure 3A). In contrast, exposure to OGD led to a more pronounced decline in cell viability (57.55 ± 2.88% mean ± SEM; n = 4). However, after 24 h of reperfusion, cell viability increased to approximately 72% (72.12 ± 2.88% cell viability, mean ± SEM; n = 4) (Figure 3B).

The five tested compounds exhibited potent neuroprotective effects, reversing the O-R/IR-induced decrease in cell viability (decline in metabolic activity) in a dose-dependent manner at all or most of the concentrations. Interestingly, **ChN2** and **QN23** demonstrated the strongest neuroprotective effects against O-R toxicity (Figure 3A), even at the lowest concentration of 0.001 μM. In the IR model, **ChN2** and **QN23** displayed a broad range of efficacy (0.1–1000 μM), although their neuroprotective effects at the higher concentrations seemed to be lower than those of the rest of the compounds (Figure 3B).

**Figure 4:**
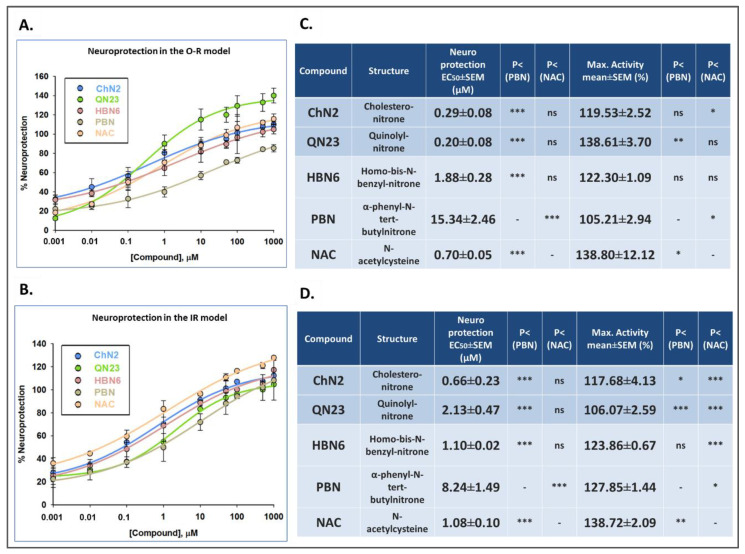
**Neuroprotective effects, effective dose 50 (EC50) values, and maximal activities of ChN2, QN23, HBN6, PBN, and NAC against loss of metabolic activity induced by (A,C) O-R treat-ment and (B,D) IR exposure in SH-SY5Y cells.** (**A**,**B**) Concentration–response curves showing % neuroprotection of the compounds at specified concentrations. The curves were fitted using non-linear weighted regression analysis to estimate the EC50 and maximal neuroprotective activity (MNA) values. Data represent the means ± SEMs of four experiments performed in triplicate. (**C**,**D**) EC50 and MNA values of the tested compounds. Statistical comparisons were made com-paring the compounds to PBN and NAC. * *p* < 0.05, ** *p* < 0.01, and *** *p* < 0.001; ns: no statisti-cally significant difference (one-way ANOVA + Holm–Sidak post hoc test).

To further explore the pharmacodynamic parameters of the tested compounds, we determined their EC_50_ and maximal neuroprotective activity (MNA) values as indicators of their efficacy and potency (Figure 4). The EC_50_ values of the compounds against O-R toxicity from the lowest to the highest were **QN23** ≤ **ChN2** ≤ **NAC** ≤ **HBN6** <<< **PBN** (Figure 4A,C). These data show that in the oxidative stress model, **ChN2** and **QN23** have similar EC_50_ values to those of **HBN6** and **NAC** and significantly lower values than **PBN**; thus, **PBN** displays the lowest efficacy of all the tested drugs. On the other hand, the MNA values showed the following order: **NAC** = **QN23** ≥ **HBN6** ≥ **ChN2** ≥ **PBN**, implying the absence of statistically significant differences between the potencies of the five assayed compounds. On the other hand, under IR conditions (Figure 4B,D), the EC_50_ values, from the lowest to the highest, follow the order **ChN2** ≤ **NAC** ≤ **HBN6** ≤ **QN23** <<< **PBN**, showing again that **ChN2**, **QN23**, **HBN6**, and **NAC** have similar EC_50_ values and significantly lower values than **PBN**. The comparison of the MNA values shows the following order of potency: **NAC** > **PBN** ≥ **HBN**6 > **ChN2** > **QN23**, indicating that under the conditions of the ischemia–reperfusion model, **ChN2** and **QN23** are the least effective compounds in terms of their maximal neuroprotective activities. All these results confirm the efficacies of **ChN2** and **QN23** at low concentrations, while their capacities to enhance cell viability decrease at high concentrations. They also show that they are effective and potent drugs, particularly against oxidative stress toxicity.

#### 3.2.2. Effects on Necrotic Cell Death

**Figure 5:**
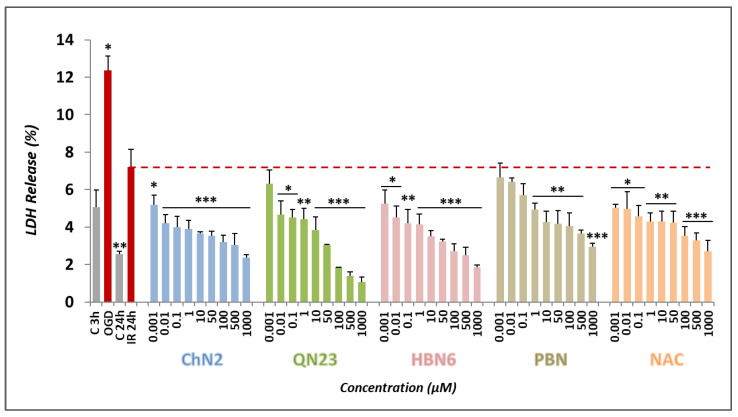
**Anti-necrotic effects of ChN2, QN23, HBN6, PBN, and NAC after the exposure of SH-SY5Y cells subjected to OGD (3 h) and oxygen and glucose resupply (OGD-R), indicated as IR (24 h).** The bars represent the % of lactate dehydrogenase (LDH) released in the presence of the tested compounds at the indicated concentrations. Data are presented as means ± SEMs of four experi-ments performed in triplicate. Statistical analyses were performed employing a one-way ANOVA + Holm–Sidak post hoc test; treatment versus IR 24 h (* *p* < 0.05; ** *p* < 0.01; *** *p* < 0.001).

To further explore the neuroprotective effects of the tested compounds, we decided to examine their anti-necrotic capacities. Necrosis is characterized by cellular swelling, the rupture of intracellular organelles, and ultimately, plasma membrane breakdown [29]. Lactate dehydrogenase (LDH), a soluble cytosolic enzyme, easily crosses the damaged cell membrane. The comparison of its activity in the culture media to its intracellular activity makes it possible to infer the magnitude of necrotic death after cell exposure to IR conditions [27]. As shown in Figure 5, OGD cell exposure induced a percentage of LDH release of about 12% (12.35 ± 0.79%, mean ± SEM; n = 4), with a recovery of up to 7% after 24 h of reperfusion, while under basal conditions, the value of LDH release did not exceed 5.5% (5.07 ± 0.92% for C3h and 2.54 ± 0.18% for C24h, mean ± SEM; n = 4). All the assayed compounds significantly decreased the release of LDH in a dose-dependent manner except for the lowest concentrations of **QN23** (0.001 μM) and **PBN** (0.001–0.1 μM) (Figure 5).

**Figure 6:**
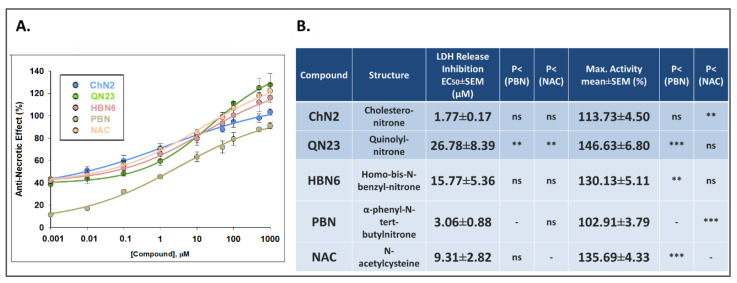
**Anti-necrotic effects of ChN2, QN23, HBN6, PBN, and NAC after IR exposure of SH-SY5Y cells.** (**A**) Dose–response curves indicating the % anti-necrotic effect values of the compounds at the specified concentrations. The curve adjustments to estimate the EC_50_ and MNA were carried out via a non-linear, ponderated regression analysis of minimal squared using logistic curves f1 = min + (max − min)/(1 + (x/EC50)^(−Hillslope)). Data represent the means ± SEMs of four experi-ments, each performed in triplicate. The analysis was implemented using SigmaPlot v.11 (Systat Sofware INC., 2012). (**B**) EC_50_ and MNA values of the tested compounds. Statistical comparisons of all values were made against **PBN** and **NAC** at ** *p* < 0.01 and *** *p* < 0.001 (one-way ANOVA). ns: no statistically significant difference.

Concentration–response curves ranging from 0.001 to 1000 μM were fitted to a non-linear regression to calculate the EC_50_ and MNA values (Figure 6). The EC_50_ values ranked the compounds from higher potency to lower potency as follows: **ChN2** ≤ **PBN** ≤ **NAC** ≤ **HBN6** < **QN23** (Figure 6B). According to their MNA values, the compounds showed this order in terms of efficacy: **QN23** ≥ **NAC** ≥ **HBN6** > **ChN2** ≥ **PBN** (Figure 6B). Consequently, **ChN2** had a similar EC_50_ value to those of **HBN6**, **PBN**, and **NAC** and a significantly lower value than the values of **QN23**. **QN23** displayed the highest MNA value, similar to the values of **HBN6** and **NAC** and significantly higher than those of **ChN2** and **PB**N. These results underscore the remarkable anti-necrotic effects of **ChN2** at low concentrations and **QN23** at high concentrations. 

#### 3.2.3. Effects on Apoptotic Cell Death

**Figure 7:**
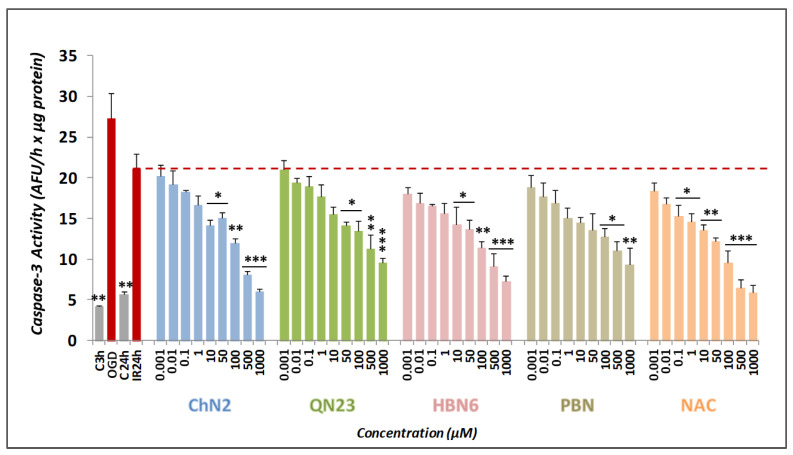
**Effect of ChN2, QN23, HBN6, PBN and NAC against apoptotic cell death induced by ische-mia-reperfusion in SH-SY5Y cells.** The bars show the Caspase-3 activity (ΔAFU/min/μg protein) in the presence of the assayed compounds at the indicated concentrations. Values represented are the mean ± SEM of five experiments, each one performed in triplicate. The statistical analyses (one-way ANOVA followed by Holm–Sidak analysis as a post-hoc test) show significant differ-ences in Caspase-3 activity against the IR condition alone at * *p* < 0.05, ** *p* < 0.01 and *** *p* < 0.001. AFU = arbitrary fluorescent units.

To comprehensively investigate the neuroprotective effects of the tested compounds, we examined their anti-apoptotic properties. Caspases—specifically Caspase-3—are the main proteins involved in this type of cell death in mammals [30]. To assess Caspase-3 activity, we used Ac-DVED-AMC, a substrate with a specific amino acid sequence that is hydrolyzed by Caspase-3, forming AMC, a fluorescent product that can be measured in the spectrofluorometer. As depicted in Figure 7, exposure to OGD resulted in a Caspase-3 activity of 27.33 ± 3.13 AFU/h × μg protein (mean ± SEM; n = 5), which decreased to 21.10 ± 1.90 (mean ± SEM; n = 5) after reperfusion. Under the basal conditions C3h and C24h, the activity was 4.21 ± 0.14 (mean ± SEM; n = 5) and 5.69 ± 0.70 AFU/h × μg (mean ± SEM; n = 5), respectively. Although the lowest concentrations (0.001 and 0.01 μM) of the assayed compounds did not exhibit significant anti-apoptotic effects, the five drugs demonstrated some degree of anti-apoptotic activity at higher doses (Figure 7). **ChN2** and **NAC** had statistically significant neuroprotective effects across a wide range of concentrations (1–1000 and 0.1–1000 μM, respectively). **QN23** and **HBN6** exhibited anti-apoptotic effects at concentrations of 50–1000 μM, while **PBN** demonstrated anti-apoptotic activity only at 100, 500 and 1000 μM (Figure 7).

**Figure 8:**
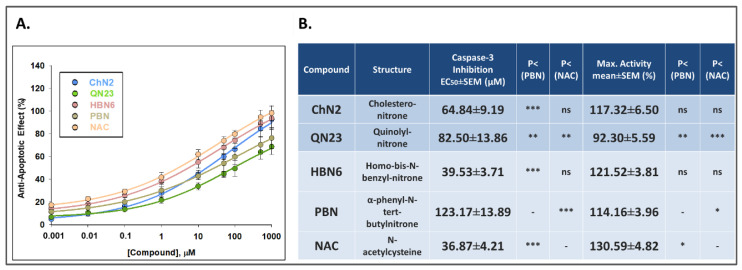
**Anti-apoptotic effects of ChN2, QN23, HBN6, PBN and NAC after IR exposure of SH-SY5Y human neuroblastoma cells.** (**A**) Dose-response curves indicating the % anti-apoptotic effect of the compounds at the indicated concentrations. The curve adjustments to estimate the EC_50_ and MNA values were carried out by non-linear ponderated regression analysis of minimal squared, using logistic curves f1 = min + (max − min)/(1 + (x/EC50)^(−Hillslope)). Data represent the mean ± SEM of five experiments, each one performed in triplicate. The analysis was implemented using SigmaPlot v.11 (Systat Sofware INC., 2012). (**B**) EC_50_ and MNA values of the assayed compounds. Statistical comparisons of all values were made against **PBN** and **NAC** at * *p* < 0.05, ** *p* < 0.01, and *** *p* < 0.001 (one-way ANOVA). ns: no statistically significant difference.

Concentration-anti-apoptotic effect curves were fitted to a non-linear regression to calculate the EC_50_ and MNA values to rank the compounds for their potency and efficacy in preventing apoptotic cell death (Figure 8). The order of EC_50_ values, from highest to lowest anti-apoptotic capacity, was as follows: **NAC** ≤ **HBN6** < **ChN2** ≤ **QN23** < **PBN** (Figure 8B). As for their MNA values, the compounds showed the following potency order: **NAC** ≥ **HBN6** ≥ **ChN2** ≥ **PBN** > **QN23** (Figure 8B). Thus, **ChN2** showed a similar MNA value to **HBN6**, **PBN** and **NAC** and, although it had a higher EC_50_ value than **NAC** and **HBN6**, it still was significantly lower than that of **PBN**. On the other hand, **QN23** displayed a EC_50_ value comparable to that of **ChN2**, but it also showed the lowest MNA value out of the five compounds. Therefore, **ChN2** exhibited pronounced anti-apoptotic properties at medium to high concentrations, while **QN23** showed less efficacy against this type of cell death, although it surpasses its precursor nitrone, **PBN**. 

#### 3.2.4. Antioxidant Effects

**Figure 9:**
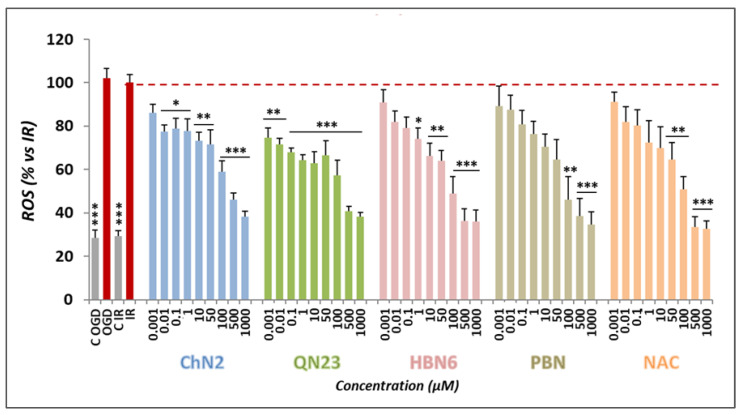
**Antioxidant effects of ChN2, QN23, HBN6, PBN and NAC in SH-SY5Y cells after IR exposure.** The bars show the % superoxide O_2_^•−^ formed after OGD (3 h) and reperfusion (2.5 h) in the pres-ence of the tested compounds at the indicated concentrations. Values represented are the mean ± SEM of four experiments, each one performed in triplicate. The statistical analyses compare the effect of IR vs. controls and the effect of the different compounds vs. the IR condition alone at * *p* < 0.05, ** *p* < 0.01 and *** *p* < 0.001 (one-way ANOVA followed by Holm–Sidak analysis as a post-hoc test).

To assess the impact of the tested compounds’ antioxidant properties on their neuroprotective capacity, we examined the rate of ROS production in SH-SY5Y cells following exposure to OGD (3 h) and reperfusion (2.5 h). To measure superoxide radical anion (O_2_^•−^) levels, we used dihydroethidium (DHE) as a fluorogenic probe. As shown in Figure 9, O_2_^•−^ production after IR (100 ± 9.29%, mean ± SEM; n = 4) was similar to O_2_^•−^ production after 3 h of OGD exposure (102.18 ± 10.68%, mean ± SEM; n = 4). **ChN2** and **QN23** were effective at most or all the tested concentrations while **HBN6**, **NAC** and, especially, **PBN**, displayed a narrower range of potency. However, they showed better concentration-dependence and more significant antioxidant effects at the highest concentrations (100–1000 μM) (Figure 9).

**Figure 10:**
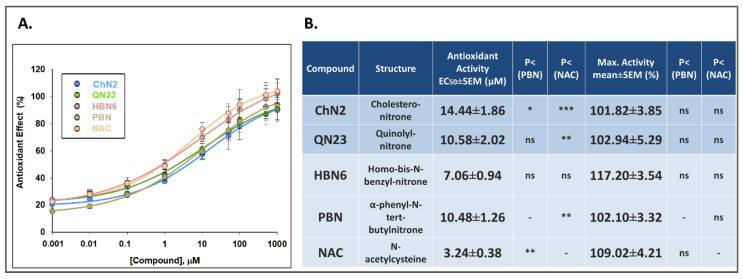
**Antioxidant effects of ChN2, QN23, HBN6, PBN and NAC after the exposure of SH-SY5Y cells to IR conditions.** (**A**) Dose-response curves indicating the % antioxidant effect of the compounds at the specified concentrations. The curve adjustments to estimate the EC_50_ and maximal antioxidant activity (MAA) were carried out by non-linear ponderated regression analysis of minimal squared, using logistic curves f1 = min + (max − min)/(1 + (x/EC50)^(−Hillslope)). Data represent the mean ± SEM of four experiments, each one performed in triplicate. The analysis was imple-mented using SigmaPlot v.11 (Systat Sofware INC., 2012). (**B**) EC_50_ and MNA values of the tested compounds. Statistical comparisons of all values were made against **PBN** and **NAC** at * *p* < 0.05, ** *p* < 0.01, and *** *p* < 0.001 (one-way ANOVA). ns: no statistically significant difference.

Concentration-antioxidant response curves and the EC_50_ values and the maximal antioxidant activities (MAA) of the tested compounds are shown in Figure 10. The order of EC_50_ values, from higher to lower antioxidant potency, was as follows: **NAC** ≤ **HBN6** ≤ **PBN** ≤ **QN23** ≤ **ChN2** (Figure 10B). Regarding the MAA values, the order of potency was as follows: **HBN6** ≥ **NAC** ≥ **QN23** ≥ **PBN** ≥ **ChN2** (Figure 10B). Notably, there were no statistically significant differences among the EC_50_ and MAA values of the five assayed compounds. 

### 3.3. Correlation Analyses between the Different Neuroprotective Properties of **ChN2**, **QN23**, **HBN6**, **PBN** and **NAC**

**Figure 11:**
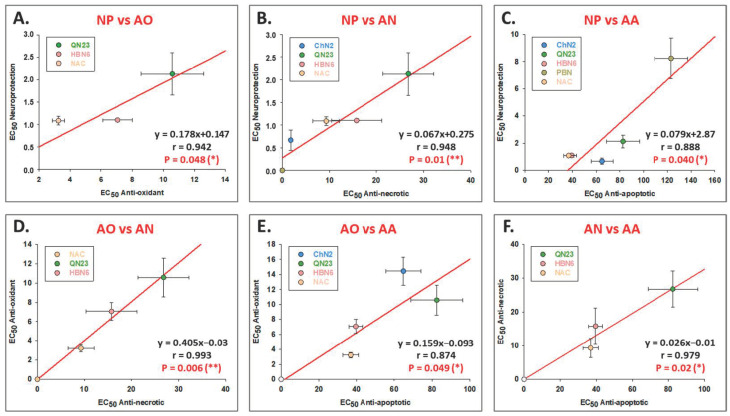
**Correlation analyses between the EC_50_ values (μM) for the neuroprotective (NP), antioxidant (AO), anti-necrotic (AN) and anti-apoptotic (AA) activities of the tested compounds.** Linear re-gression analysis shows correlation between (**A**) NP and AO, (**B**) NP and AN, (**C**) NP vs. AA, (**D**) AO and AN, (**E**) AO and AA, (**F**) AN and AA. Straight line equations (ax + b), correlation coefficients (r) and the statistical significance of regression analyses are indicated in each plot. Regression analyses and statistics were performed by the Pearson Correlation Test at * *p* < 0.05 and ** *p* < 0.01, using SigmaPlot v.11 (Systat Sofware INC., 2012).

To establish a comparative relationship between the different types of neuroprotective activities of the compounds and determine their contributions to neuroprotection, we performed correlation analyses. Individual analyses with data from all nine concentrations (0.001–1000 μM) of each compound indicated a statistically significant correlation between all neuroprotective mechanisms. We conducted linear correlation between the neuroprotective activity and the anti-apoptotic, anti-necrotic or antioxidant effects for all the compounds, comparing their EC_50_ to be able to group them according to their mechanisms of action (Figure 11). First, we observed a correlation between the neuroprotective and antioxidant activity of **QN23**, **HBN6** and **NAC** (Figure 11A). Moreover, the anti-necrotic effects of these three compounds were also significantly correlated with their antioxidant (Figure 11D) and anti-apoptotic (Figure 11F) capacity. **ChN2**, **QN23**, **HBN6** and **NAC** showed a significant correlation between their neuroprotective and anti-necrotic activity (Figure 11B) and between their antioxidant and anti-apoptotic activity (Figure 11E). Finally, we observed a clear correlation between the neuroprotective and anti-apoptotic effects of the five studied compounds (Figure 11C). These results indicate that, while the antioxidant, anti-necrotic and anti-apoptotic effects of **QN23**, **HBN6** and **NAC** seem to be equally relevant for their overall neuroprotective capacity, **ChN2**’s protective properties seem to derive especially from its anti-necrotic and anti-apoptotic activity, and **PBN** depends mainly on its anti-apoptotic capacity. 

## 4. Discussion

The present study aimed to delve deeper into the analysis of the neuroprotective and antioxidant mechanisms of two synthetic nitrones, cholesteronitrone 2 (**ChN2**) and quinolylnitrone 23 (**QN23**), which have shown promising therapeutic potential for the treatment of ischemic stroke in previous in vitro and in vivo studies [4,18,20,22]. Our objectives were to expand the knowledge of their neuroprotective effects at a wider range of concentrations and against two of the main types of cell death involved in ischemia, namely, apoptosis and necrosis. Notably, the anti-necrotic effects of these nitrones were previously unexplored. Additionally, we aimed to compare the protective and antioxidant capacities of **ChN2** and **QN23** with those of **HBN6**, another nitrone studied by our research group [10], as well as **PBN** and **NAC**, which were used as standards. 

In order to do so, we decided to use cultures of the human neuroblastoma cell line SH-SY5Y as our study model. While experiments conducted using cell lines have their limitations as they do not fully replicate the physiological characteristics of a real biological system, they also offer several advantages. Cell line models are accessible, relatively easy to maintain, provide a readily available and abundant supply of cells, and allow for control over all environmental variables, thereby enhancing reproducibility [26,31]. In fact, human in vitro systems are commonly employed for cost-effective high-throughput screening assays to assess the efficacy of therapeutic compounds [26,31]. The SH-SY5Y cell line in particular is a well-established model frequently employed in neuroprotection studies related to ischemic stroke [25,26] and neurodegenerative diseases [27,28].

Our findings revealed that **ChN2** and **QN23** had basal neurotoxic effects at high concentrations (100–1000 μM), which was not observed with any of the reference compounds (Figure 2). Nevertheless, these two nitrones also showed remarkable neuroprotective potencies in oxidative stress and ischemia–reperfusion models (Figure 3 and Figure 4). **ChN2** and **QN23** both displayed a wide range of efficacy (0.001–1000 μM) under both the O-R treatment and the OGD-R models. While they both exhibited exceptional effectiveness in increasing cell viability at high concentrations in the oxidative stress model, their efficacies were not as high in the IR model (Figure 3 and Figure 4). These results were further supported by the statistical analysis of the EC_50_ and MNA values.** ChN2** (EC_50_ = 0.29 ± 0.08 μM) and **QN23** (EC_50_ = 0.20 ± 0.08 μM) were, at low concentrations, the most neuroprotective compounds against oxidative stress (Figure 4). **ChN2** also exhibited a notable potency in the ischemia model (EC_50_ = 0.66 ± 0.23 μM), while **QN23** (EC_50_ = 2.13 ± 0.47 μM) displayed a slightly higher EC_50_ value, although it was significantly lower than that of **PBN** (EC_50_ = 8.24 ± 1.49 μM) (Figure 4). On the other hand, **ChN2** and **QN23** were the compounds with the lowest MNA values, which can be attributed to their demonstrated basal neurotoxicities at high concentrations (also observed in other works, such as that of Ayuso et al. in 2015) [18]. However, it should be noted that the observed MNA values are higher—and therefore denote greater neuroprotective efficacies—than the values previously reported for many other nitrones investigated by our research group. As an example, **HBN1** showed an MNA value of 68.55 ± 3.96%, while **ChN2** and **QN23** always reached values greater than 100% [10]. Therefore, the results obtained in this study underscore the notable neuroprotective capacities of **ChN2** and **QN23**, particularly at lower concentrations, which holds significant implications at the clinical level since much lower doses of **ChN2** and **QN23** would be needed to achieve the same therapeutic effects when compared to other nitrones.

Next, we aimed to investigate the contributions of the anti-necrotic and anti-apoptotic effects of **ChN2** and **QN23** to their neuroprotective effects. Both nitrones exhibited notable anti-necrotic properties, with **ChN2** demonstrating a wide range of efficacy (0.001–1000 μM), while **QN23** displayed the highest protective capacity against necrotic cell death at the highest tested concentrations (100–1000 μM) (Figure 5). In terms of their EC_50_ values, **ChN2** (EC_50_ = 1.77 ± 0.17 μM) showed a similar value to **HBN6**, **PBN**, and **NAC**, while its MNA value (MNA = 113.73 ± 4.50%) was significantly lower than that of **HBN6** and **NAC** but comparable to **PBN** (Figure 6). Conversely, **QN23** had the highest EC_50_ value (EC_50_ = 26.78 ± 8.39 μM) among all tested compounds yet displayed a similar MNA value (MNA = 146.63 ± 6.80%) to those of **HBN6** and **NAC** and a significantly higher value than **PBN** (Figure 6). Regarding their anti-apoptotic effects, both **ChN2** and **QN23**, along with the three standards, showed more limited efficacies. **ChN2** and **QN23** did not have neuroprotective effects against apoptosis at the lowest concentrations (0.001–10 μM), which was the same as **HBN6** (Figure 7). However, **ChN2** seemed to be as effective as **NAC** at the highest concentrations tested (500 and 1000 μM). Both **ChN2** and **QN23** displayed higher EC_50_ values (EC_50_ = 64.84 ± 9.19 and 82.50 ± 13.86, respectively) than **HBN6** and **NAC** but significantly lower values than that of **PBN** (Figure 8). Interestingly, the EC_50_ values of the anti-apoptotic effects in this study were significantly higher compared to previous studies conducted by our research group (e.g., **PBN** and **NAC** had EC_50_ values of 1.12 ± 0.27 μM and 0.05 ± 0.01 μM, respectively) [21]. This discrepancy could be attributed to several factors, including the use of lower concentrations of the compounds in this study (as **ChN2** and **QN23** demonstrated very promising results at 0.001 and 0.01 μM on the cell viability test), which may have impacted the shapes of the concentration–response curves, as well as the use of a different batch of cells, which may have introduced additional variability. In terms of the MNA value, **ChN2** exhibited a similar value (MNA = 117.32 ± 6.50%) to **HBN6**, **PBN**, and NAC, while QN23 displayed the lowest MNA activity (MNA = 92.30 ± 5.59%) among all the tested compounds.

When evaluating the antioxidant properties of **ChN2** and **QN23**, we found that they both showed a wide range of effectiveness. They notably had the widest ranges of efficacy, with **QN23** being able to reduce O_2_^•−^ production at all the tested concentrations (Figure 9). These results are consistent with previous findings from our research group [18,20]. Notably, both nitrones showed similar EC_50_ and MAA values to those of **HBN6**, **PBN**, and **NAC** (Figure 10). However, they displayed better antioxidant profiles than other previously studied synthetic nitrones, such as **HBN3** (EC_50_ = 38.52 ± 5.25 μM) and **QN2** (MAA= 82.22 ± 5.37%), with **ChN2** and **QN23** exhibiting EC_50_ values below 15 μM and both compounds surpassing 100% activity [10,21].

Our final objective was to determine the contribution of different neuroprotective mechanisms to the overall neuroprotective capacity of each compound. Correlation analyses indicated that the neuroprotective effects of **ChN2** were particularly dependent on its anti-necrotic and anti-apoptotic activities. In the case of **QN23**, **HBN6**, and **NAC**, their protective capacities seemed to be equally due to anti-necrotic, anti-apoptotic, and antioxidant capacities. Conversely, the neuroprotection provided by **PBN** was primarily dependent on its anti-apoptotic activity (Figure 11). It is worth noting that these results are consistent with the maximal activity data, which were not included in the correlation analyses. Notably, the MNA values of **ChN2** for its anti-necrotic (MNA = 113.73 ± 4.50%) and anti-apoptotic (MNA = 117.62 ± 6.50%) effects significantly exceeded its MAA values (101.82 ± 3.85%). Conversely, the maximal anti-apoptotic activity of **PBN** (MNA = 114.16 ± 3.96%) greatly surpasses its MNA values associated with its anti-necrotic and anti-apoptotic properties (MNA = 102.91 ± 3.79% and MNA = 102.10 ± 3.32%, respectively). One possible explanation for the difference in the neuroprotective mechanisms of **ChN2** and **QN23** can be found in their structural differences. **ChN2** is a steroid nitrone with a cholesterol skeleton, while **QN23** is a nitrone inserted into a quinoline heterocyclic ring. Additionally, **ChN2** bears an N-methyl substituent at the nitrogen of the nitrone motif, whereas **QN23** features a tert-butyl substituent at the nitrogen of the nitrone functional group.

In conclusion, **ChN2** and **QN23** demonstrated significant antioxidant, anti-necrotic, and anti-apoptotic properties. We examined their effects across a broad range of concentrations, which is particularly valuable as it allows for the determination of the optimal concentrations to be employed in future study models (e.g., organoids) or when investigating their impact on other types of cell death associated with cerebral ischemia (autophagy, pyroptosis, and ferroptosis) [11,12], mitochondrial membrane potential, and ATP production. Such investigations could provide deeper insight into the mechanisms underlying the neuroprotective effects of these compounds, elucidating the factors influencing their distinct neuroprotective activities.

## 5. Conclusions

Here, we conducted a comprehensive examination of the neuroprotective profiles of **ChN2** and **QN23**, two synthetic nitrones that have demonstrated considerable potential for the treatment of ischemic stroke in previous in vitro and in vivo studies conducted by our research group. Our aim was to extend the existing knowledge by investigating their neuroprotective and antioxidant mechanisms across a wider range of concentrations, comparing them with three standard compounds (**HBN6**, **PBN** and **NAC**), and exploring their impacts on necrosis and apoptosis, which had not been previously explored. 

In conclusion, our findings highlight the promising therapeutic potential of **ChN2** and **QN23** as neuroprotective agents, particularly at low concentrations. Notably, **ChN2** and **QN23** displayed significant anti-necrotic properties, acted as effective antioxidant molecules, and exhibited anti-apoptotic effects, albeit with more limited efficacies and only at high concentrations. Furthermore, **ChN2**’s effect primarily relied on its anti-necrotic and anti-apoptotic activities for neuroprotection, while **QN23**’s protective capacity against necrosis and apoptosis was equally relevant to its antioxidant effects. These findings underscore the potential of **ChN2** and **QN23** as promising candidates for the treatment of ischemic stroke, as their neuroprotective effects are evident even at low concentrations. The elucidation of their mechanisms of action and their comparison with other nitrones contribute to a better understanding of their efficacy and position them as viable candidates for further exploration and potential clinical applications.

## Data Availability

The data presented in this study are available upon request from the corresponding author.

## References

[1] Paul S., Candelario-Jalil E. (2021). Emerging neuroprotective strategies for the treatment of ischemic stroke: An overview of clinical and preclinical studies. Exp. Neurol..

[2] Qin C., Yang S., Chu Y.H., Zhang H., Pang X.W., Chen L., Zhou L.Q., Chen M., Tian D.S., Wang W. (2022). Signaling pathways involved in ischemic stroke: Molecular mechanisms and therapeutic interventions. Signal Transduct. Target. Ther..

[3] Rodrigo R., Fernández-Gajardo R., Gutiérrez R., Matamala J.M., Carrasco R., Miranda-Merchak A., Feuerhake W. (2013). Oxidative Stress and Pathophysiology of Ischemic Stroke: Novel Therapeutic Opportunities. CNS Neurol. Disord. Drug. Targets.

[4] Martínez-Alonso E., Escobar-Peso A., Ayuso M.I., Gonzalo-Gobernado R., Chioua M., Montoya J.J., Montaner J., Fernández I., Marco-Contelles J., Alcázar A. (2020). Characterization of a CholesteroNitrone (ISQ-201), a Novel Drug Candidate for the Treatment of Ischemic Stroke. Antioxidants.

[5] Brouns R., De Deyn P.P. (2009). The complexity of neurobiological processes in acute ischemic stroke. Clin. Neurol. Neurosurg..

[6] Dirnagl U., Iadecola C., Moskowitz M.A. (1999). Pathobiology of ischaemic stroke: An integrated view. Trends Neurosci..

[7] Love S. (1999). Oxidative stress in brain ischemia. Brain Pathol..

[8] Chan P.H. (2001). Reactive oxygen radicals in signaling and damage in the ischemic brain. J. Cereb. Blood Flow Metab..

[9] Latour L.L., Kang D.W., Ezzeddine M.A., Chalela J.A., Warach S. (2004). Early blood-brain barrier disruption in human focal brain ischemia. Ann. Neurol..

[10] Chamorro B., Diez-Iriepa D., Merás-Sáiz B., Chioua M., García-Vieira D., Iriepa I., Hadjipavlou-Litina D., López-Muñoz F., Martínez-Murillo R., González-Nieto D. (2020). Synthesis, antioxidant properties and neuroprotection of α-phenyl-tert-butylnitrone derived HomoBisNitrones in in vitro and in vivo ischemia models. Sci. Rep..

[11] Abbruzzese G., Morón-Oset J., Díaz-Castroverde S., García-Font N., Roncero C., López-Muñoz F., Marco Contelles J.L., Oset-Gasque M.J. (2020). Neuroprotection by Phytoestrogens in the Model of Deprivation and Resupply of Oxygen and Glucose In Vitro: The Contribution of Autophagy and Related Signaling Mechanisms. Antioxidants.

[12] Chen D.-Q., Guo Y., Li X., Zhang G.-Q., Li P. (2022). Small molecules as modulators of regulated cell death against ischemia/reperfusion injury. Med. Res. Rev..

[13] Tymianski M. (2013). Novel Approaches to Neuroprotection Trials in Acute Ischemic Stroke. Stroke.

[14] Goenka L., Uppugunduri Satyanarayanab C.R., Kumar S., George M. (2019). Neuroprotective agents in Acute Ischemic Stroke—A Reality Check. Biomed. Pharmacother..

[15] Savitz S.I., Baron J.C., Fisher M. (2019). Stroke Treatment Academic Industry Roundtable X: Brain Cytoprotection Therapies in the Reperfusion Era. Stroke.

[16] Lyden P., Buchan A., Boltze J., Fisher M. (2021). Top Priorities for Cerebroprotective Studies-A Paradigm Shift: Report From STAIR XI. Stroke.

[17] Samadi A., Soriano E., Revuelta J., Valderas C., Chioua M., Garrido I., Bartolomé B., Tomassolli I., Ismaili L., González-Lafuente L. (2011). Synthesis, structure, theoretical and experimental in vitro antioxidant/pharmacological properties of a-aryl, N-alkylnitrones, as potential agents for the treatment of cerebral ischemia. Bioorg. Med. Chem..

[18] Ayuso M.I., Chioua M., Martínez-Alonso E., Soriano E., Montaner J., Masjuán J., Hadjipavlou-Litina D.J., Marco-Contelles J., Alcázar A. (2015). CholesteroNitrones for Stroke. J. Med. Chem..

[19] Chioua M., Sucunza D., Soriano E., Hadjipavlou-Litina D., Alcázar A., Ayuso A., Oset-Gasque M.J., González M.P., Monjas L., Rodríguez-Franco M.I. (2012). α-Aryl-N-alkyl nitrones, as potential agents for stroke treatment: Synthesis, theoretical calculations, antioxidant, anti-inflammatory, neuroprotective and brain-blood barrier permeability properties. J. Med. Chem.

[20] Chioua M., Martínez-Alonso E., Gonzalo-Gobernado R., Ayuso M.I., Escobar-Peso A., Infantes L., Hadjipavlou-Litina D., Montoya J.J., Montaner J., Alcázar A. (2019). New Quinolylnitrones for Stroke Therapy: Antioxidant and Neuroprotective (Z)-N-tert-Butyl-1-(2-chloro-6-methoxyquinolin-3-yl)methanimine Oxide as a New Lead-Compound for Ischemic Stroke Treatment. J. Med. Chem..

[21] Chamorro B., Izquierdo-Bermejo S., Serrano J., Hadjipavlou-Litina D., Chioua M., López-Muñoz F., Marco-Contelles J., Martínez-Murillo R., Oset-Gasque M.J. (2023). Neuroprotective and antioxidant properties of new quinolylnitrones in in vitro and in vivo cerebral ischemia models. Sci. Rep..

[22] Martínez-Alonso E., Escobar-Peso A., Aliena-Valero A., Torregrosa G., Chioua M., Fernández-Serra R., González-Nieto D., Ouahid Y., Salom J.B., Masjuan J. (2022). Preclinical Characterization of Antioxidant Quinolyl Nitrone QN23 as a New Candidate for the Treatment of Ischemic Stroke. Antioxidants.

[23] Diez-Iriepa D., Chamorro B., Talaván M., Chioua M., Iriepa I., Hadjipavlou-Litina D., López-Muñoz F., Marco-Contelles J., Oset-Gasque M.J. (2020). Homo-Tris-Nitrones Derived from α-Phenyl-N-tert-butylnitrone: Synthesis, Neuroprotection and Antioxidant Properties. Int. J. Mol. Sci..

[24] Piotrowska D.G., Mediavilla L., Cuarental L., Głowacka I.E., Marco-Contelles J., Hadjipavlou-Litina D., López-Muñoz F., Oset-Gasque M.J. (2019). Synthesis and Neuroprotective Properties of N-Substituted C-Dialkoxyphosphorylated Nitrones. ACS Omega.

[25] Chien-Hung L., Nicol C.J.B., Cheng Y.-C., Yen C., Wang Y.-S., Chiang M.-C. (2020). Neuroprotective effects of resveratrol against oxygen glucose deprivation induced mitochondrial dysfunction by activation of AMPK in SH-SY5Y cells with 3D gelatin scaffold. Brain Res..

[26] Holloway P.M., Gavins F.N. (2016). Modeling ischemic stroke in vitro: Status quo and future perspectives. Stroke.

[27] Xie H.R., Hu L.S., Li G.Y. (2010). SH-SY5Y human neuroblastoma cell line: In vitro cell model of dopaminergic neurons in Parkinson’s disease. Chin. Med. J..

[28] Kruger T.M., Bell K.J., Lansakara T.I., Tivanski A.V., Doorn J.A., Stevens L.L. (2019). Reduced Extracellular Matrix Stiffness Prompts SH-SY5Y Cell Softening and Actin Turnover To Selectively Increase Aβ(1–42) Endocytosis. ACS Chem. Neurosci..

[29] Chan F.K.-G., Moriwaki K., De Rosa M.J. (2013). Detection of necrosis by release of lactate dehydrogenase activity. Methods Mol. Biol..

[30] Porter A.G., Jänicke R.U. (1999). Emerging roles of caspase-3 in apoptosis. Cell Death Differ..

[31] Pradillo J.M., García-Culebras A., Cuartero M.I., Peña-Martínez C., Moro M.A., Lizasoain I., Moraga A. (2022). Del laboratorio a la clínica en el ictus isquémico agudo. Modelos experimentales in vitro e in vivo. Rev. Neurol..

